# Lipotoxicity is glucose-dependent in INS-1E cells but not in human islets and MIN6 cells

**DOI:** 10.1186/1476-511X-10-115

**Published:** 2011-07-11

**Authors:** Ernest Sargsyan, Peter Bergsten

**Affiliations:** 1 Department of Medical Cell Biology, Uppsala University, Box 571, SE-75123, Uppsala, Sweden

**Keywords:** beta-cell, human islets, lipotoxicity, apoptosis, palmitate oxidation

## Abstract

**Background:**

Prolonged elevated levels of lipids have negative effects on beta-cell function and mass (lipotoxicity). To what extent exposure to high glucose concentration is important in the harmful effects of lipids (glucolipotoxicity) has been debated.

**Methods:**

We addressed beta-cell lipotoxicity by measuring apoptosis in isolated intact control human islets and insulin-secreting cell lines MIN6 and INS-1E cultured in the presence of palmitate and low (5.5 mM) or high (25 mM) glucose for 48 hours.

**Results:**

In both cell lines and human islets palmitate induced apoptosis after culture at low glucose. Palmitate-induced apoptosis was not increased after culture at high compared to low glucose in human islets and MIN6 cells but glucose-induced rise in apoptosis was observed in INS-1E cells. The rise in apoptosis in INS-1E cells was partially reversed by inclusion of AMPK-agonist AICAR. When CPT1-inhibitor etomoxir was included during culture at low glucose palmitate-triggered apoptosis was accentuated both in the islets and the cell lines. Palmitate oxidation in human islets and the cell lines was comparable after culture at low glucose. At high glucose, palmitate oxidation was reduced by 30% in human islets and MIN6 cells but by 80% in INS-1E cells. In INS-1E cells, AICAR increased oxidation of palmitate. Presence of etomoxir at low glucose decreased palmitate oxidation both in the islets and the cell lines.

**Conclusions:**

In summary, lipotoxicity is evident not only in the presence of high but also low glucose concentrations. Additional effects of glucose are prominent in INS-1E but not in MIN6 cells and intact control human islets, which are able to efficiently oxidize fatty acids at high glucose and in this way avoid glucolipotoxicity.

## Introduction

Elevated blood glucose and lipid levels are manifestations of individuals with type 2 diabetes mellitus [1-4]. Insulin-secreting beta-cells in these individuals are characterized by impaired glucose-stimulated insulin secretion (GSIS) and enhanced apoptosis [5]. Several mechanisms of such toxicity have been proposed including ceramide formation [6], increased oxidative stress [7] and activation of ER stress [8]. However, it remains debated whether elevated fatty acids may impair beta-cell function and cause apoptosis at normal glucose concentrations (lipotoxicity) or only in combination with elevated glucose levels (glucolipotoxicity). It has been suggested that hyperlipidemia alone is not detrimental for beta-cells [9,10]. Hyperglycemia was proposed to be a prerequisite for fatty acid induced beta-cell dysfunction and death. Beta-cells exposed to combined high levels of glucose and fatty acids preferentially metabolize glucose [11] and direct long chain (LC)-CoA towards toxic non-oxidative metabolic pathways [12,13]. Lipid oxidation is inhibited by elevated levels of glucose-derived malonyl-CoA, which regulate lipid partitioning through its inhibitory action on mitochondrial fatty acid transporter carnitine palmitoyltransferase-1 (CPT-1) [14]. Consistent with these ideas it was shown that AICAR and metformin, agents that favor fatty acid oxidation prevent lipotoxicity whereas etomoxir, an agent that inhibits β-oxidation, exerts opposite effects [11,15].

Analysis of studies performed on intact human islets indicates that fatty acids exert their toxic effect independently of glucose concentration [6,16,17]. Also, *in vivo *studies showed that sustained increase in plasma free fatty acids impairs insulin secretion in non-diabetic subjects genetically predisposed to develop T2DM [18].

In the present study we have addressed beta-cell lipotoxicity by measuring how glucose affects palmitate-induced apoptosis in isolated human islets and insulin-producing cell lines INS-1E and MIN6. Palmitate is an abundant fatty acid present in the circulation and particularly linked with development of beta-cell dysfunction [19,20]. Based on the malonyl-CoA long-chain hypothesis [14] we evaluated to what extent high glucose levels inhibited lipid oxidation as a mechanism of beta-cell glucolipotoxicity. The results indicate that palmitate provokes its negative effect even at low glucose. High glucose accentuates palmitate-induced apoptosis in INS-1E cells but not in human islets and in MIN6 cells. We propose that this is due to limited glucose-induced inhibition of fatty acid oxidation in human islets and MIN6 cells.

## Materials and methods

### Cell culture

Rat INS-1E cells (a kind gift from Dr. Pierre Maechler, Geneva University) were cultivated in RPMI 1640 medium containing 11 mM glucose and supplemented with 10% fetal bovine serum (FBS), 2 mM L-glutamine, 1 mM sodium pyruvate, 10 mM HEPES and 55 μM β-mercaptoethanol at 37°C and 5% CO_2_. All reagents were purchased from Invitrogen (Carlsbad, CA). Experiments with INS-1E cells were performed between passages 65 and 90. Mouse insulinoma MIN6 cells (a kind gift from Prof. Jun-Ichi Miyazaki, Osaka University) were maintained in Dulbecco's Modified Eagle medium (DMEM) containing 25 mM glucose and supplemented with 10% FBS and 55 μM β-mercaptoethanol at 37°C and 5% CO_2_. All experiments with MIN6 cells were performed between passages 21 and 28. Human islets were obtained from the Islet Transplantation Unit at Uppsala University from non-diabetic individuals. Human islets were cultured in CMRL 1066 medium containing 5.5 mM glucose and supplemented with 10% FBS.

### Ethics Statement

Ethical permission to use human islets isolated from healthy individuals have been obtained from the Regional Ethical Review Board in Uppsala (date: 2010-02-10; number 2010/006).

### Free fatty acid preparation and cell/islet treatment

Culture medium containing palmitate (Sigma, St. Louis, MO) was prepared as previously described [8,21]. Briefly, the fatty acid was dissolved in 50% ethanol to a concentration of 100 mM. This stock solution was diluted in culture medium to a required concentration and then allowed to complex with 0.5% fatty acid free BSA (Boehringer Mannheim GmbH, Mannheim, Germany) for 30 min at 37°C. Cells cultured to 65-70% confluence or ~50 human islets were exposed to palmitate in the presence of different concentrations of glucose for 48 hours. Whereas FBS was maintained during palmitate exposure of INS-1E and MIN6 cells [8,22], FBS was removed during palmitate incubation of human islets [16]. Cells and islets cultured in the presence of palmitate were also treated with 1 mM AICAR or 0.2 mM etomoxir (both purchased from Sigma).

### Measurements of palmitate oxidation rate and accumulation

Cells/islets were cultured in media containing 0.5 mM palmitate and 2 μCi [^3^H]palmitate per ml. After culture, media samples were collected and ^3^H_2_O separated from [^3^H]palmitate using Folch extraction [23]. To measure accumulation, cells and islets were lysed using H_2_O. The volume of 10 ml Ultima Gold™ scintillation fluid (Chemical Instruments AB, Sollentuna, Sweden) was added to 500 μl ^3^H_2_O and radioactivity determined by a liquid-scintillation spectrometer (Wallac System 1400™ PerkinElmer, Boston, MA). The results were normalized to DNA content.

### Measurements of metabolic activity

Metabolic activity was determined by MTT (Sigma Aldrich) assay, which measures NAD(P)H production through glycolysis [24]. MTT solution was prepared as 5 mg/ml stock in PBS just before use. After culture, 50 μl of MTT solution was added to each well. After 4 hours incubation, medium containing MTT was removed and 500 μl of DMSO added to the cells. The spectrophotometric absorbance was then measured at 540 nm. The results were normalized to DNA content.

### Protein measurements by Western blot analysis

Cells/islets were washed twice with PBS and then lysed in 1% Triton X100 in PBS containing protease inhibitor mixture (Sigma) for 30 min. After lysis, cells were scraped and centrifuged at 12,000 rpm for 10 min. Ensuing supernatant was collected, and total protein concentration was determined by the DC protein assay (Bio-Rad, Hercules, CA) according to the manufacturer's instruction. Equal amounts of protein were solubilized by SDS-PAGE sample buffer, boiled for 5 min, resolved by SDS-PAGE and transferred to PVDF membrane. Immunoblot analyses were performed with antibodies towards cleaved form of caspase 3, phosphorylated acetyl-CoA carboxylase (p-ACC), ACC and fatty acid synthase (FAS) (all from Cell Signaling, Beverly, MA). Immunoreactive bands were visualized with Fluor-S MultiImager MAX (Bio-Rad) and quantified with Quantity One software (Bio-Rad). To normalize the expression level of each protein, PVDF membranes were stained with Coomassie, scanned and quantified with the Quantity One software.

### Apoptosis measurements by DNA fragmentation analysis

Fragmentation of DNA in cells/islets was assayed with the cell death detection kit ELISA^PLUS ^(Roche Diagnostics, Mannheim, Germany) according to the manufacturer's instructions. Twenty islets of comparable size were used for measurements [6]. The ELISA measures cytoplasmic oligonucleosomes that increase after apoptosis-associated DNA degradation. The measurements were related to DNA content.

### Data analysis

Results are presented as means ± SEM. Statistical significance between two conditions was analyzed using one-way ANOVA with Tukey post-hoc test. *P *< 0.05 was considered statistically significant.

## Results

### Palmitate-induced apoptosis is accentuated by high glucose in INS-1E cells but not in human islets and MIN6 cells

**Figure 1:**
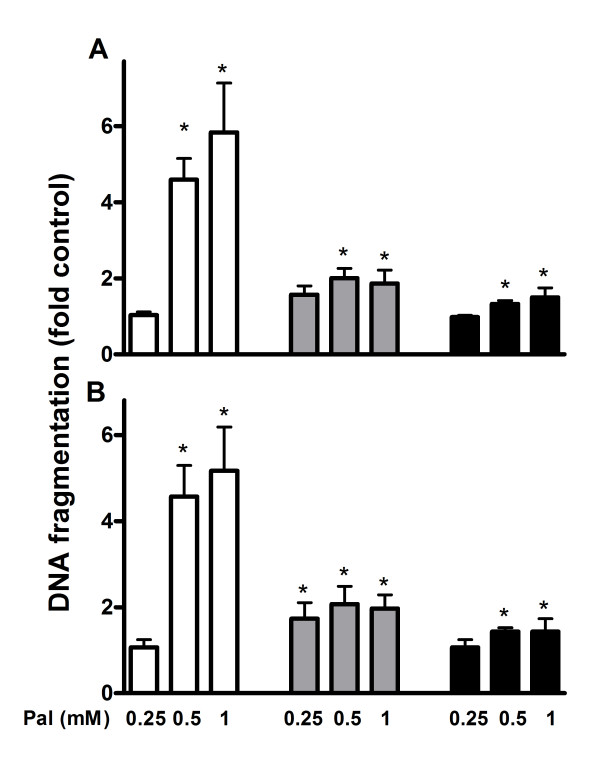
**Apoptosis in palmitate-exposed INS-1E cells (white bars), MIN6 cells (grey bars) and human islets (black bars) measured as DNA fragmentation**. The cells and islets were cultured for 24 (A) and 48 (B) hours in the presence of 0.25, 0.5 and 1 mM palmitate. INS-1E cells cultured at 11 mM glucose, MIN6 cells at 25 mM glucose and human islets at 5.5 mM glucose alone were considered as controls. Results are means ± SEM of 3-4 independent experiments. *P < 0.05 compared to controls.

**Figure 2:**
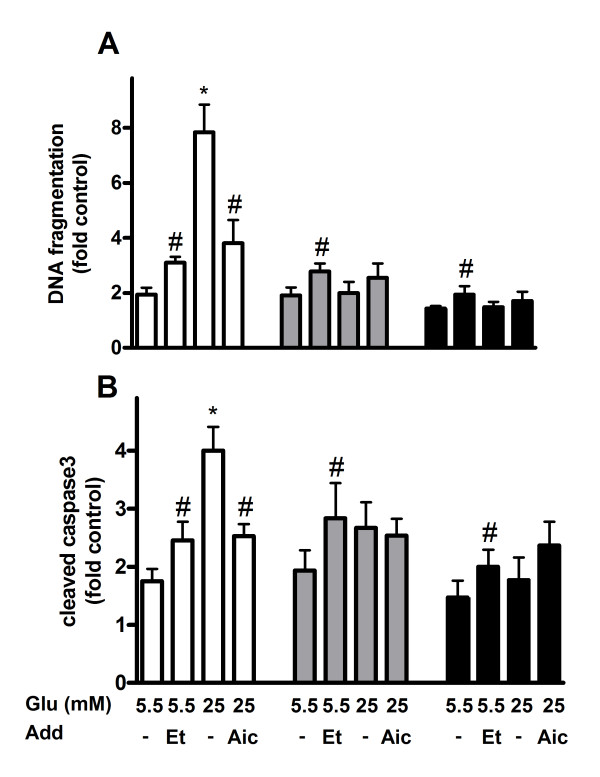
**Apoptosis in palmitate-exposed INS-1E cells (white bars), MIN6 cells (grey bars) and human islets (black bars) measured as DNA fragmentation (top panel) and cleaved caspase 3 (bottom panel)**. The cells and islets were cultured for 48 hours in the presence of 0.5 mM palmitate at indicated glucose (Glu) concentrations. AICAR (1 mM; Aic) and etomoxir (0.2 mM; Et) were added as indicated. INS-1E cells cultured at 11 mM glucose, MIN6 cells at 25 mM glucose and human islets at 5.5 mM glucose alone were considered as controls. Results are means ± SEM of 4-6 independent experiments. *P < 0.05 compared to low glucose and #p < 0.05 effect of additive.

Apoptosis was measured after culturing INS-1E and MIN6 cells and human islets in the presence of 0.25, 0.5 and 1 mM palmitate for 24 and 48 hours. Elevated levels of apoptosis were observed in the cell lines and human islets, when the ambient palmitate concentration was 0.5 mM or higher and the culture time was 24 hours (Figure 1A) or 48 hours (Figure 1B). Palmitate-induced apoptosis was more accentuated in INS-1E cells compared to MIN6 and human islets. We next examined to what extent the glucose concentration affected palmitate-induced apoptosis. Whereas DNA fragmentation was 8-fold and expression of active caspase 4-fold in palmitate-treated INS-1E cells exposed to high (25 mM) glucose compared to control cells, these parameters were changed only ~2-fold in INS-1E cells exposed to low (5.5 mM) glucose (Figure 2). Apoptosis was 2-fold increased in MIN6 cells and 1.5-fold in human islets irrespective of glucose concentrations i.e. no differences in apoptosis were observed when palmitate-exposed MIN6 cells and human islets were cultured at low or high glucose. To elaborate the role of fatty acid oxidation, AICAR and etomoxir were used. When AICAR was added to cells exposed to palmitate and high glucose, DNA fragmentation and caspase 3 levels observed in INS-1E cells were lowered compared to palmitate treatment alone. AICAR-treatment did not affect apoptosis in human islet or MIN6 cells, however. When etomoxir was added to cells exposed to palmitate and low glucose ~1.5-fold rise in the levels of DNA fragmentation and cleaved caspase 3 was observed in the cell lines and islets compared to palmitate treatment alone.

### Palmitate oxidation is substantially reduced by high glucose in INS-1E cells but only moderately in human islets and MIN6 cells

**Figure 3:**
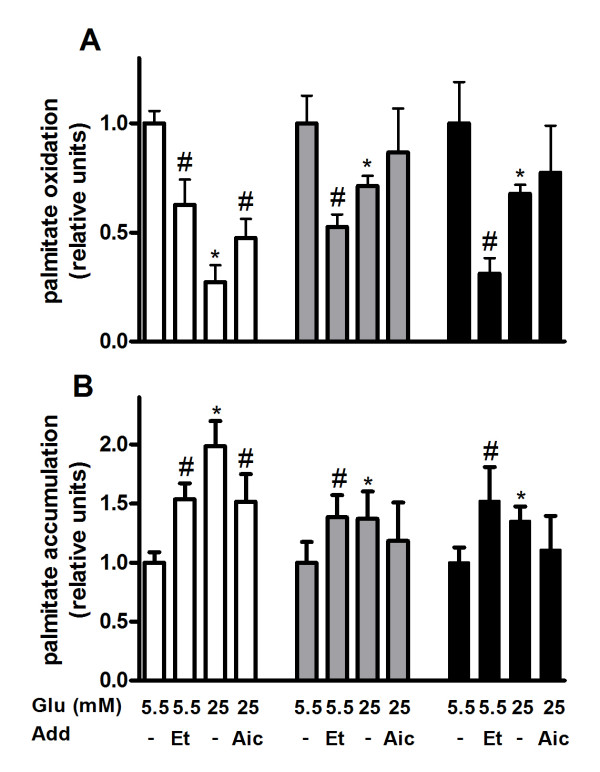
**Palmitate oxidation (A) and accumulation (B) in palmitate-exposed INS-1E cells (white bars), MIN6 cells (grey bars) and human islets (black bars)**. The cells and islets were cultured for 48 hours in the presence of 0.5 mM palmitate and 2 μCi [^3^H]palmitate per ml at indicated glucose (Glu) concentrations. AICAR (1 mM; Aic) and etomoxir (0.2 mM; Et) were added as indicated. After culture, media were collected, ^3^H_2_O separated and radioactivity determined by a liquid-scintillation spectrometer. To measure palmitate accumulation, radioactivity was determined in lysed cells and islets. Results are means ± SEM of 3-4 independent experiments. *P < 0.05 compared to low glucose and #p < 0.05 effect of additive.

**Figure 4:**
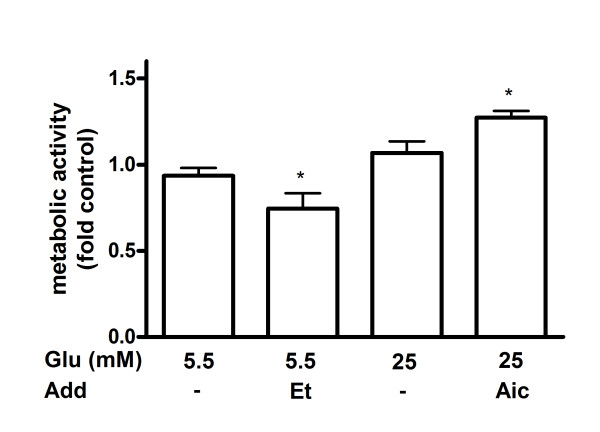
**Metabolic activity in palmitate-exposed INS-1E cells**. Cells were cultured for 48 hours in the presence of 0.5 mM palmitate at indicated glucose (Glu) concentrations. AICAR (1 mM; Aic) and etomoxir (0.2 mM; Et) were added as indicated. After culture, metabolic activity was measured using the MTT assay. INS-1E cells cultured at 11 mM glucose alone were considered as control. Results are means ± SEM of 3 independent experiments. *P < 0.05 compared to control.

The glucose-dependency in INS-1E cells and glucose-independency in human islets and MIN6 cells of palmitate-induced apoptosis (Figure 2) was hypothesized to depend on differences in oxidation of the fatty acid. Palmitate oxidation was therefore measured in palmitate-exposed INS-1E and MIN6 cells and human islets cultured at high or low glucose for 48 hours. In INS-1E cells exposed to palmitate oxidation rate of the fatty acid was reduced by 80% in cells cultured at high glucose compared to cells cultured at low glucose (Figure 3A). Glucose-dependent palmitate oxidation was also seen in MIN6 cells and human islets although to a much less extent. In human islets and MIN6 cells cultured at high glucose palmitate oxidation was lowered by about 30% compared to islets and cells cultured at low glucose. Next, we compared oxidation rates of palmitate in palmitate-exposed INS-1E cells, MIN6 cells and human islets after 48 hours. At high glucose human islets oxidized the fatty acid two times more than MIN6 cells and four times more than INS-1E cells (Figure 3A). In contrast, no significant difference in palmitate oxidation was observed between palmitate-exposed human islets, MIN6 and INS-1E cells cultured at low glucose. Addition of AICAR restored the glucose-dependent reduction in palmitate oxidation partially in INS-1E cells but not in MIN6 cells and human islets (Figure 3A). When etomoxir was added palmitate oxidation was reduced both in cell lines and islets. The reduction was especially pronounced in human islets (Figure 3A). Measurements of palmitate accumulation were in agreement with oxidation data. Higher oxidation rate of palmitate was accompanied by lower accumulation of palmitate inside the cells/islets (Figure 3B). To exclude the possibility that the pronounced glucose-induced difference in palmitate oxidation observed in INS-1E cells was due to changes in metabolic activity we measured mitochondrial activity in these cells after 48 hours culture in the presence of palmitate and normalized it to DNA content. As expected, activity was reduced by etomoxir and induced by AICAR but was not altered by changing the glucose concentration (Figure 4).

### ACC activity is substantially enhanced by high glucose in INS-1E cells but only moderately in human islets and MIN6 cells

**Figure 5:**
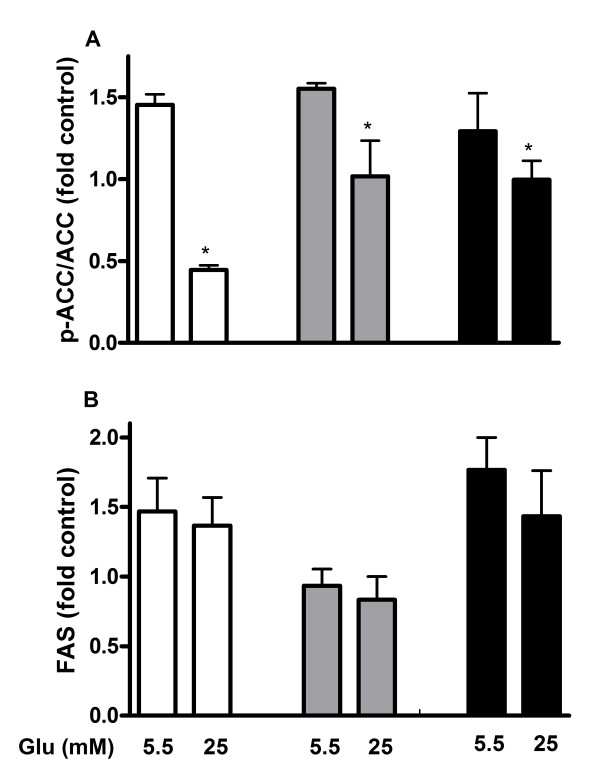
**p-ACC/ACC ratio (A) and FAS expression level (B) in palmitate-exposed INS-1E cells (white bars), MIN6 cells (grey bars) and human islets (black bars)**. The islets and cells were cultured for 48 hours in the presence of 0.5 mM palmitate at indicated glucose (Glu) concentrations. INS-1E cells cultured at 11 mM glucose, MIN6 cells at 25 mM glucose and human islets at 5.5 mM glucose were considered as controls. After culture, protein levels of p-ACC, ACC and FAS were measured. Results are means ± SEM of 3-4 independent experiments. *P < 0.05 compared to control.

Oxidation rate of palmitate is regulated by malonyl-CoA, which inhibits CPT1 and prevents translocation of fatty acids into the mitochondria. The cellular pool of malonyl-CoA is determined by opposing activities of ACC and FAS. To address the role of malonyl-CoA in glucose-regulated oxidation of palmitate expression levels of p-ACC, ACC and FAS were measured in INS-1E cells, MIN6 cells and human islets exposed to palmitate in the presence of 5.5 and 25 mM glucose. In INS-1E cells p-ACC/ACC was reduced by 70% at high glucose compared to low glucose indicating higher activity of ACC and higher production of malonyl-CoA in the presence of high glucose (Figure 5A). In MIN6 cells and human islets the ratio was only reduced by 20-30% at high glucose compared to low glucose. When FAS was examined in cells and islets no significant difference was observed when compared with effects of palmitate at high and low glucose (Figure 5B).

## Discussion

Augmented blood lipid and glucose levels are characteristics of type 2 diabetes mellitus [1,2]. The simultaneously elevated levels of nutrients have been proposed to play a key role in the development of beta-cell toxicity manifested by impaired GSIS and reduced mass [9,10]. In the present study beta-cell lipotoxicity was evaluated by measuring beta-cell apoptosis. We demonstrated that palmitate has a toxic effect at low glucose concentration. Accentuation of fatty-acid induced apoptosis by high glucose was observed in INS-1E cells but not in human islets and MIN6 cells.

In many studies addressing beta-cell glucolipotoxicity insulin-producing cell lines were used [14,22,25,26]. In agreement with previous reports, enhanced apoptosis was observed in the present study when INS-1E cells were exposed to glucolipotoxic conditions. In contrast, no glucolipotoxic effect was observed in MIN6 cells and, more importantly, in human islets. Evidence that intact human islets do not show signs of glucolipotoxicity can also be found in other studies [6,16,17]. In contrast, when human islets were dispersed, enhanced palmitate-induced apoptosis was observed when the glucose concentration was increased [11,25,26]. It appears that intact islet architecture is protective against the combined effects of palmitate and high glucose. Along these lines, superior performance of the intact islet compared to dispersed islet cells has been demonstrated with regard to insulin secretion [27].

The negative effects on the beta-cell of simultaneous exposure to elevated levels of fatty acids and glucose have been proposed to depend on reduced fatty acid oxidation due to inhibition of CPT1 by glucose-induced formation of malonyl-CoA [13,14]. In support of this notion the negative effects of palmitate on beta-cell apoptosis has been demonstrated to be accentuated if the fatty acid is directed away from oxidative pathways by pharmacological inhibition of CPT1 in some [11,22] but not all [28] studies. To test the malonyl-CoA hypothesis, etomoxir was added during culture in the present study. Reduction of fatty acid oxidation was paralleled by increased apoptosis in both cells and islets, reinforcing the role of palmitate oxidation in lipotoxicity. Formation of ceramide and other pro-apoptotic lipid species has been proposed as underlying cause for the rise in beta-cell apoptosis observed when palmitate oxidation is reduced [6]. In this context it should be noted that triacylglycerol (TAG) formation may represent a less harmful way for the cell to channel surplus fatty acids [28].

The fact that FAS was not altered in the present study may indicate that lipogenesis and the formation of TAG is not activated in palmitate-treated beta-cells which is in agreement with a recent paper where beta-cells exposed to palmitate showed much lower oxidation rate and TAG formation than cells exposed to oleate [29]. Also, FAS and other enzymes of lipogenesis controlled by SREBP-1c are activated much more when palmitate is replaced by oleate [29]. At the same time, accumulation of radioactivity inside the cell/islets indicates that palmitate is directed towards formation of other species rather than TAG.

Whereas high glucose concentration reduced palmitate oxidation to a minor extent in human islets, the reduction was accentuated in INS-1E cells. We conclude that human islets obtained from control donors are able to efficiently oxidize fatty acids at high glucose and in this way avoid glucolipotoxicity. Substantial glucose-induced reduction in palmitate oxidation in INS cells was also observed in a previous study [30]. The observed difference in palmitate oxidation might be explained by higher ACC activity in INS-1E cells compared to human islets. The glucose-induced reduction in p-ACC observed in the present study is almost identical to the reduction in palmitate oxidation, which could be explained by a rise in malonyl-CoA [14]. The observation that differences in glucose-induced changes in ACC activity correspond with changes in palmitate oxidation but not fully with changes in apoptosis illustrates the complexity by which palmitate influences the beta-cell. Our results also indicate that beta-cell mitochondrial function is not affected to any major extent in the presence of palmitate during the indicated time periods since mitochondrial activity was not changed much when taking into account apoptotic cells/islets.

The observation that palmitate-induced apoptosis is evident not only in the presence of high but also low glucose concentrations underscores the importance of not only maintaining normal blood glucose but also lipid levels in individuals with T2DM. Also, in obese individuals, especially the ones with a genetic background of T2DM, normalizing blood lipid levels would be extremely important to prevent beta-cell apoptosis. Whether the onset of diabetes and elevated glucose levels in obese individuals accelerates beta-cell destruction is not completely clear from this study. The enhanced palmitate-induced apoptosis at high glucose observed in INS-1E cells but not in islets obtained from healthy individuals and in MIN6 cells might be explained by limited inhibition of fatty acid oxidation by malonyl-CoA in the latter and may speak in favor of the notion that glucolipotoxicity becomes manifest given a genetic background predisposing to beta-cell failure.

## Conclusion

We conclude that lipotoxicity is evident not only in the presence of high but also low glucose concentrations. It underscores the importance to not only maintaining normal blood glucose but also lipid levels in individuals with T2DM. Our data also suggest that healthy human islets are able to efficiently oxidize fatty acids at high glucose and in this way avoid glucolipotoxicity.

## Competing interests

The authors declare that they have no competing interests.

## Authors' contributions

ES participated in the design of the study, carried out all the studies, analyzed the data and drafted the manuscript. PB participated in the study design and helped to draft the manuscript. All authors have read and approved the final manuscript.
